# Supplementary material for: A population-based serological survey of Vibrio cholerae antibody titers in Ouest Department, Haiti in the year prior to the 2022 cholera outbreak
Source: medRxiv. 2023 Feb 8:2023.02.06.23285537. Preprint. [Version 1] doi: 10.1101/2023.02.06.23285537 (PMC9934795; doi:10.1101/2023.02.06.23285537)
Supplement: Supplement 1 [file media-1.pdf]

Supplemental Table 1. Descriptive Statistics for Age Comparisons

Category	Median	Range	25th Percentile	75th Percentile	*p* value
**LPS IgG**					
≥5 years old	50.00	107.9	41.62	59.87	
<5 years old	43.73	66.11	34.60	54.88	**<0.0001**
4 years old	35.36	41.87	30.66	52.66	**0.0341**
3 years old	43.73	36.82	34.49	55.23	0.1018
2 years old	54.14	50.58	35.73	58.31	0.9633
1 year old	43.77	62.41	34.80	53.59	0.0595
<1 year old	33.96	37.12	28.65	42.03	**<0.0001**
**LPS IgA**					
≥5 years old	15.15	64.89	11.19	20.85	
<5 years old	8.075	19.43	6.038	11.20	**<0.0001**
4 years old	9.675	17.97	6.628	13.52	**0.0052**
3 years old	8.770	12.18	7.140	13.16	**0.0003**
2 years old	7.940	12.59	5.590	9.955	**<0.0001**
1 year old	7.175	19.43	4.930	9.928	**<0.0001**
<1 year old	3.075	9.100	1.953	3.730	**<0.0001**
**CtxB IgG**					
≥5 years old	39.12	106.0	31.55	49.02	
<5 years old	45.11	83.92	36.78	55.85	**0.0033**
4 years old	45.77	23.11	37.98	49.23	0.9937
3 years old	38.23	45.07	33.87	48.46	0.9993
2 years old	53.30	75.26	38.50	66.82	**0.0024**
1 year old	44.22	75.73	36.96	60.15	**0.0011**
<1 year old	34.01	50.40	24.36	46.60	0.4507
**CtxB IgA**					
≥5 years old	15.50	78.58	10.81	23.08	
<5 years old	13.95	72.12	8.620	19.77	**0.0138**
4 years old	14.09	31.46	8.738	21.62	0.8780
3 years old	15.75	72.12	8.520	22.76	0.9979
2 years old	14.03	40.64	8.278	21.80	0.9999
1 year old	12.50	55.40	8.265	17.38	0.7348
<1 year old	4.900	11.41	1.440	6.470	**0.0004**

Supplemental Table 1. Descriptive Statistics for Age Comparisons. The median, interquartile range and p-value for each of the age comparisons is shown. Children younger than 5 years were compared in aggregate to children and adults 5 years old and older using an unpaired two-tailed student t test. Individual age groups of 1, 2, 3, and 4-years were compared to older children and adults by one-way ANOVA. Units for all columns excluding the p-value refer to ELISA units.

Supplemental Figure 1. ELISA Units analyzed by age of participant.

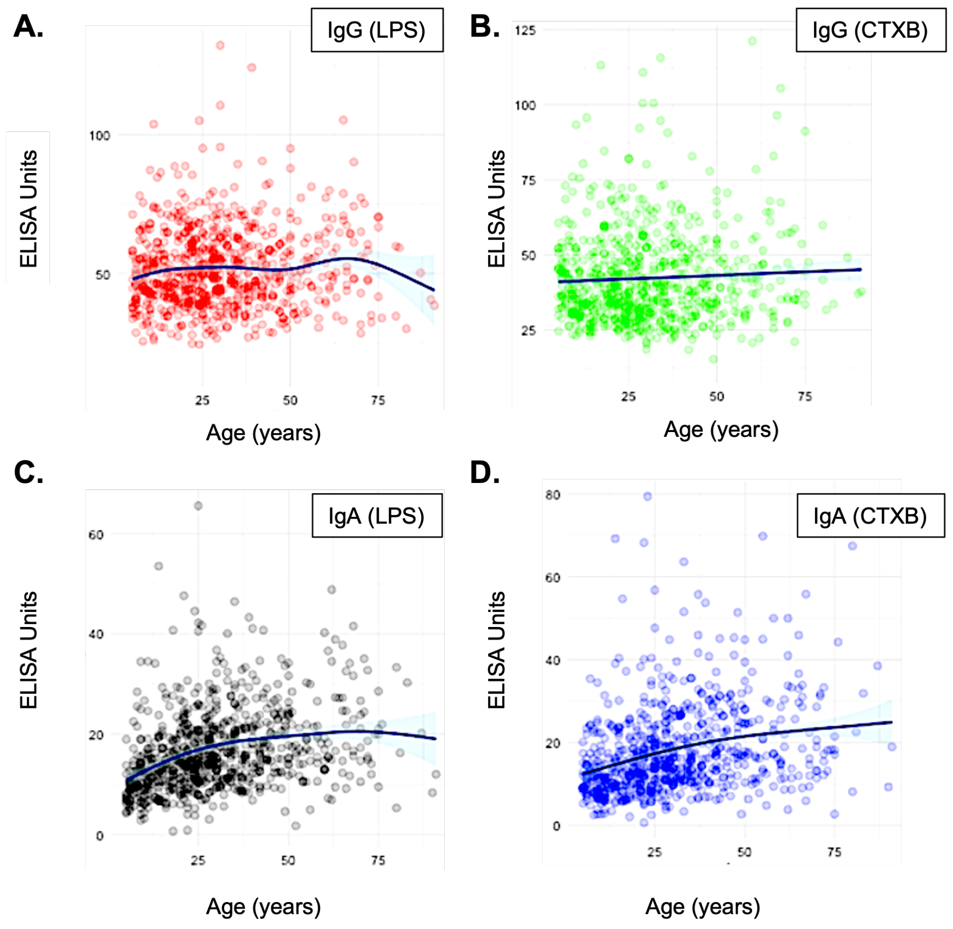

Supplemental Figure 1. Antibody levels expressed in ELISA units analyzed by age of participant using a generalized additive model (GAM). **A.** IgG to *V. cholerae* LPS (effective degrees of freedom, EDF, 4.9, p=0.12); **B.** IgG to *V. cholerae* CtxB (EDF 1.0, p=0.13); **C.** IgA to *V. cholerae* LPS (EDF, 3.4, p<2e-16); **D.** IgA to *V. cholerae* CtxB (EDF 2.0, p<2e-16)**.** A statistically significant association (p<0.05) was identified between age and IgA for both LPS and CtxB.
